# Popular Science

**Published:** 1883-04

**Authors:** 


On another page of this journal we have arranged such a bill of fare, the general features of which, we believe, our readers will approve. It is not to be expected that every one can follow out this bill of fare to the letter, our object being only to suggest such dishes as would have the merit of variety and which could be substituted by equivalents. But in order to derive the greatest benefit from a well arranged bill of fare, it is essential that all the dishes should be properly prepared ; and to those who have not had special training or long experience in such matters we would recommend a little book published by Fords, Howard & Hulbert, New York, entitled "THE EASIEST WAY IN HOUSEKEEPING AND COOKING," by HELEN CAMPBELL. This is the most complete " cook book " we have seen, in that it gives all the details of each process in language so plain that a novice ean easily understand them.

CURES FOR BALDNESS.—The *Chemists' Journal* says : Dr. X Landerer, of Athens, has again been so obliging as to send us some notes from the cradle of pharmacy.

Numberless remedies for baldness of French, English, German and American origin stock our markets, but none, according to Dr. Landerer, equal in efficiency the following, which he has used and prescribed for many years past: Prepare a tincture of the cups of the *Quercus œgilops*, which are known in commerce as valonia, and digest with it powdered cloves and cinnamon. Make a tincture by digesting the leaves of the *Laurus apollonis* in acid wine, and mix the two together. Before applying this remedy, the skin of the head should be well washed with a decoction of saponaria root (*Saponaria levantica*), to cure any *exanthema pithyriatis* which may be present. Instead of pomatum or hair oil, laurel oil should be used, this being the usual hair oil in vogue among the ladies of the East. Dr. Landerer calls this remedy for baldness *alexitrichon*, or hair preserver.

OVERHEAD FIRE ESCAPES.—A Tasmanian correspondent suggests as a fire escape a passway of iron along and above the roofs of houses ; passing through the more lofty buildings if need be, or diverging to the right or left so as to bridge over and connect all the houses of a block, thus securing an easy and safe passage from any house to those adjacent, as well for the convenience of firemen as for the escape of those who are beset by fire. The construction of these iron passes, he says, could be fairly compulsory to owners, and they need be by no means of an unsightly appearance. When wished, they could be elegantly constructed to conform to the general architecture of the building by or through which it passes, and this would hold good with regard to the means by which each house was connected with this proposed passway. He is aware that there are disadvantages which at once crop up—apparent danger from burglars, and so on—but there is no good without its modicum of evil, and this weakness of his plans, he thinks, could be overcome and guarded against.

YOUR attention is directed to the advertisement of Caulocorea as prepared under the direction of Warren Lowell, M. D., which is decidedly the most important remedial agent ever discovered for the treatment of all those weaknesses and diseases peculiar to women. The formula of this preparation contains just those drugs which practical experience has verified to be the very best for the conditions for which it is recommended. The very large number of physicians who use Caulocorea testifies to its great value as a nerve tonic and an important therapeutic agent in diseases of the female reproductive organs. It is certainly a sedative and uterine tonic of great power, and is therefore the remedy *par excellence* in painful menstruation or threatened abortion.

AMERICAN TIN MINES.—A correspondent states that tin has been recently discovered in Alabama, under conditions which give every prospect of developing a large and paying industry. For the past two years, Mr. G. W. Gesner, of New York City, has been steadily at work on his lands, about two miles southeast of Ashland, Clay County, Alabama, erecting buildings, opening quarries, and putting up machinery preparatory to mining, washing and smelting the ores of tin found there. The property known as the Broad Arrow mines embraces nearly a square mile, and from its hilly, undulating surface is well adapted to the easiest method of mining. Mr. Gesner has now forty-five stamps working, the first attempt to work tin ore on the spot where found in the United States.

AN artificial wood for various ornamental purposes has lately been introduced in Germany with very satisfactory results. The crude mass from which the articles thus produced are pressed consists chiefly of cellulose mixed with any sort of starch. That is, the ordinary commercial cellulose, which is to be had in any quantity in the form of paper, is softened in water and thoroughly disintegrated, then put in a fine meshed sieve and the water drained off ; it is now mixed with about three parts, by weight, of dry starch, made from wheat, rye, potatoes, Indian corn, etc., as well as two parts of rye or wheat flour, or corn meal, or any other flour that contains gluten and very intimately mixed.

NEVER be witty at another's expense; it is bartering away friendship at a low price.

THE very instant yon perceive yourself in a passion shut your mouth, and keep it shut till your blood cools. This advice, if always followed, would save many a life of bitterness, and of deep, incurable anguish.

LONDON is enormous, but the statement that it contains, 5,000,000 people is apt to mislead. What is called the metropolitan area, consisting of land within a radius of fifteen miles of Charing Cross, does contain very nearly that number; but estimating in this manner from the City Hall, our metropolitan area would probably include 3,000,000. To talk, therefore, of London having 5,000,000 people is delusive.

POISONS.—For any poison, take a heaping teaspoonful of ground mustard, stirred in a glass of cold water; it causes instantaneous vomiting. If you have no mustard use common salt instead. When vomiting ceases, swallow two or three tablespoonfuls of sweet oil or other mild oil, butter or lard, which prevent the poison being absorbed into the blood: then send for the doctor.

POPULAR SCIENCE.—The following, says the *Chemical News*, is from a recent number of the *Ashton Reporter:* "Water carried as Gas.—M. Pasteur, a nephew of the celebrated chemist of that name, has recently adapted an old discovery to great practical use. It is a well-known fact that the crossing of the great African desert is accomplished by means of caravans composed of camels, horses, etc., the water for which has to be transported on the back of the consumer. This lessens to a great degree their freighting capacity. M. Pasteur has established suitable works at the numerous termini of the routes for separating the water into oxygen and hydrogen. As the latter is sixteen times lighter than the former, and is the gas used in balloons, it carries the oxygen and a considerable part of the camel, besides furnishing light on dark nights. He unites the gases by the simple means of explosion when desired for use. The French Government has created M. Pasteur a commander of the Legion of Honor for his great adaptation."

